# Novel Nitric Oxide-Releasing Formulations Show Fungicidal Potential for Superficial Dermatophyte Infection

**DOI:** 10.3390/jof12030228

**Published:** 2026-03-21

**Authors:** Aditya K. Gupta, Elizabeth A. Cooper, Lisa Miller, Morgan Kearl, Chris C. Miller, Harmanpreet Kaur, Najmeh Dorafshanian, James Martins, Simon J. L. Teskey, Jeremy Road

**Affiliations:** 1 Mediprobe Research Inc., London, ON N5X 2P1, Canada; lcooper@mediproberesearch.com; 2 Division of Dermatology, Department of Medicine, Temerty Faculty of Medicine, University of Toronto, Toronto, ON M5S 3H2, Canada; 3 Faculty of Medicine, University of British Columbia, Vancouver, BC V6T 1Z3, Canada; 4 Valley Podiatry, Chilliwack, BC V2R 0J2, Canada; 5 SaNOtize Research & Development Corp, Vancouver, BC V6P 6T3, Canadajmartins@sanotize.com (J.M.);

**Keywords:** antifungal agent, dermatophyte, nitric oxide, tinea pedis, topical therapy, onychomycosis

## Abstract

A commercially available nitric oxide (NO)-releasing solution (NORS) has demonstrated in vitro efficacy for dermatophytosis, but a NO-releasing gel (NORG) may be more suitable for patient self-application. We present a preliminary investigation of NORS for tinea pedis and an in vitro investigation of NORG for dermatophyte infection, to complement the existing published data and expand support for a possible role of NO formulations in superficial dermatophyte infection. In vitro usage of NORS and NORG is reviewed. The antifungal efficacy of NORG was assessed via time-kill assays, zone of inhibition tests with synthetic dermal membrane permeation, and scanning electron microscopy. A randomized, controlled pilot study of NORS for tinea pedis investigated the safety and efficacy of treatment over three consecutive days, with a day-31 follow-up. The NORG demonstrated rapid fungicidal activity against *T. rubrum* and *T. mentagrophytes* and effective dermal membrane penetration while retaining antifungal action. Significant morphological damage to fungal cells was noted, indicating possible fungicidal activity. The clinical NORS treatment reduced the clinical symptom severity score by 67% on average, with no significant safety findings. These findings, in addition to existing publications, support NO-releasing formulations as potential therapies that warrant further clinical investigation for superficial fungal infection.

## 1. Introduction

Superficial dermatophyte infections have been considered readily treatable for many decades since the advent of azole and allylamine development, using both topical and oral formulations. However, dermatophyte fungi are developing mechanisms to resist the antifungal activity of these drug classes, prompting a need for the development of alternative treatments [1,2].

Nitric oxide (NO) is a small endogenous diffusible molecule that readily diffuses across biological membranes and is involved in a wide variety of human physiological regulations, including immunomodulation [3,4,5,6]. NO has shown effective immunoregulatory activity and broad-spectrum antimicrobial actions, as well as biofilm dispersal properties, which have been useful in the treatment of infections such as athlete’s foot and diabetic foot wounds [7,8,9,10,11]. Antiviral effects of a NO gel have led to its approval for molluscum contagiosum by the FDA, and other widespread uses of NO-releasing compounds are being recognized within the medical field [12,13,14].

NO has also exhibited broad-spectrum antimicrobial efficacy, in vitro and in vivo, against diverse dermatological pathogens [7,15,16,17]. These findings suggest a possible role for NO-releasing products in superficial dermatophyte fungal infections.

Commercial nitric oxide-releasing formulas (www.nowonder.com) are available in the United States, including:

• NO-Releasing Solution (NORS) used as a cleansing agent, administered by foot bath.
• NO-Releasing Gel (NORG) for topical nail wellness application.

The development of commercially available NO-releasing formulas provides a source of standardized treatment for use in clinical investigation. There is existing evidence for the utility of these NO-releasing solutions, both in vitro and through clinical study, against dermatophytes [18,19,20]. We provide additional in vitro NORG evaluation for dermatophytes and clinical use of NORS for tinea pedis. These data broaden the pilot evidence of these formulas for superficial dermatophyte infection and support the development of larger-scale clinical trials for efficacy and safety.

## 2. Methods and Materials

### 2.1. In Vitro Testing with NORG

#### 2.1.1. Fungal Strains, Culture Conditions, and Antifungal Formulations

Two dermatophyte species were selected for the in vitro testing. *Trichophyton rubrum* (ATCC 28188) and *Trichophyton mentagrophytes* (ATCC 18748) were sourced from the American Type Culture Collection (ATCC; Manassas, VA, USA). The cultures were maintained in Sabouraud dextrose broth supplemented with 25% glycerol and stored at –80 °C. Routine working cultures were propagated in Sabouraud dextrose agar (SDA), incubated at temperatures ranging from 23 °C to 27 °C for seven days, and subcultured every 28 days to ensure viability.

The proprietary NORG formulation was used as the test formula. The NORG was provided by SaNOtize Inc. (Vancouver, BC, Canada). Vehicle gel was also provided for use as a placebo control where needed. The brief half-life of NO complicates the measurement of formula concentrations. The use of area-under-the-curve (AUC) measures forms the basis of concentration, and NORG provides expected NO doses of 96.2 ppm × min/cm^2^ in the 15 min following the gel dose dispensing/mixing (in-house data).

Tolnaftate was chosen as the most comparable market comparator to NORG; topical terbinafine is not widely available without a prescription, and many other standard antifungals remain prescription-only, which does not position these products in line with the marketed wellness NORG formulation.

Two tolnaftate 1% solutions [CVS Health Tolnaftate 1% Maximum Strength Anti-fungal Liquid (‘Tolnaftate’) and Tolcylen Antifungal/Nail Renewal Solution (‘Tolcylen’)] were supplied by the Center for Medical Mycology at Case Western Reserve University (Cleveland, OH, USA). Tolnaftate is an over-the-counter (OTC) antifungal product that is widely available for tinea pedis, and, as such, is readily comparable with the marketed wellness NORG formulation. Tolcylen solution provides tolnaftate in a patented low surface tension delivery vehicle designed to improve drug penetration into skin and nails versus regular tolnaftate formulas.

#### 2.1.2. Antifungal Time-Kill Assays

The fungicidal activity of NORG was evaluated via time-kill assays. The fungal suspensions of *T. rubrum* and *T. mentagrophytes* that were harvested from cultured SDA plates following a seven-day incubation at 23–27 °C were quantified through serial dilution in normal saline (0.9%) solution. Initial fungal concentrations averaged 10^6^ ± 10^1^ CFU/mL. For each assay, 0.25 g of NORG, or NORG vehicle as a control, was vortex-mixed with 1 mL fungal suspension in 15 mL centrifuge tubes. The NORG provides expected NO doses of 96.2 ppm × min/cm^2^ in the 15 min following the gel dose dispensing/mixing. Following exposure durations of 1, 2.5, 5, and 15 min, fungal suspensions were neutralized with 9 mL phosphate-buffered saline (PBS) containing 5% sodium hydroxide (1.0 N). Subsequently, 100 µL samples were plated onto SDA for enumeration post-incubation. All time points that were tested were plated and incubated for 7 days following exposure. The assay was conducted in 3 experimental replicates, each with 2 technical replicates. *t*-test comparisons for statistical significance (*p* < 0.05) of the mean eradication time of NORG versus the vehicle gel placebo were calculated using Microsoft Excel software.

#### 2.1.3. Zone of Inhibition Assays Using Synthetic Dermal Membranes

Synthetic dermal membranes (Strat-M, Millipore) simulating human dermal tissue were utilized to assess dermal penetration and antifungal efficacy. Suspensions of *T. rubrum* and *T. mentagrophytes* (0.25 mL plated containing 10^6^ CFU/mL for both species) were uniformly spread on SDA plates to establish fungal lawns, after which dermal membranes were centrally positioned and treated with antifungal agents (0.25 g of NORG, Tolcylen, Tolnaftate, or NORG vehicle). The NORG provides expected NO doses of 96.2 ppm × min/cm^2^ in the 15 min following the gel dose dispensing/mixing. The plates underwent incubation at 23–27 °C, with once daily treatment reapplication over four days, followed by an additional four-day incubation period. The zones of inhibition were subsequently measured. The experiment was conducted with 4 experimental repeats, each with two technical replicates. The *t*-test comparisons for the statistical significance (*p* < 0.05) of the average zone of inhibition measure of NORG versus the alternate treatments were calculated using Microsoft Excel software for assays with measured zones of inhibition.

#### 2.1.4. Scanning Electron Microscopy (SEM)

SEM was employed to visually elucidate the antifungal effects of NORG on *T. rubrum*. The fungal cultures were exposed to 0.25 g NORG across synthetic dermal membranes in a Franz diffusion cell setup. A parallel Franz diffusion cell setup was used to review untreated fungal cultures as a control. Post-24 h exposure, samples were processed with glutaraldehyde fixation and subsequently analyzed at the Bio-Imaging Facility, University of British Columbia, Canada.

### 2.2. In Vivo Pilot Use of No-Releasing Solution for Tinea Pedis

A single-center, double-blind, randomized, controlled exploratory pilot study was conducted in Vancouver, Canada. Adults with clinically moderate tinea pedis and a clinical symptom severity score (CSSS) of ≥20 were randomized 2:1 to receive treatment with NORS or tap water (placebo) on 3 consecutive days. The study was performed following approval by the IntegReview independent human research ethics board (REB) and Health Canada (HC6-24-c195535). Written informed consent for study participation and photo use was obtained from all participants.

Although tinea pedis may have been on both feet, only the single most severe lesion area on one foot was identified, treated, and evaluated at each visit. The participants received either the NORS or a placebo (tap water) in the form of a 5 L warm (32–37 °C) foot bath and were instructed to immerse the entire foot for 30 min. The NORS was prepared and packaged in a separate laboratory and prepared for treatment with warm tap water in a separate room at the clinic (both investigator and participant were blind to assigned treatment group). After 30 min, the participant removed their foot from the footbath and allowed it to air dry. New cotton socks and insoles were provided. Each participant in each group received three 30 min foot baths on 3 consecutive days in the clinic. The total exposure time to active treatment or placebo intervention was 90 min. The participants returned to the clinic on days 17 and 31 for follow-up evaluations. Prophylactic measures against reinfection were requested of patients during the following period, which included continued use of the new insoles, wearing clean socks, not walking barefoot, and drying the feet/interdigital spaces thoroughly after washing.

The participant baseline and treatment symptom responses were captured by the CSSS, which scores each of 8 symptoms on an 8-point scale: fissures/cracks, yellow crusting (ruptured blisters), maceration (white moist skin), scaling, erythema, vesicles/blisters, pruritus (itching), and burning, for a maximum score of 64. Dermal scrapings for mycological culture and potassium hydroxide (KOH) microscopic evaluation were obtained on treatment days 1 and 3 and at follow-up visits on days 17 and 31; mycology testing was performed by an independent laboratory (LifeLabs Medical Laboratory Services BC, Vancouver, BC, Canada).

The efficacy was evaluated by the number of participants with a complete total cure, defined as a complete mycological cure (as determined by a negative fungal culture and a negative microscopic evaluation via KOH preparation), and a complete clinical cure, defined as a CSSS < 9 at day 31. Statistical comparison of the mean CSSS between the NORS and the placebo groups was made on days 1, 3, 17 and 31 using two-way ANOVA testing (significance level of *p* < 0.05) and Microsoft Excel software. Due to the 7- to 14-day mycology testing process, subjects were enrolled, and treatment was completed prior to the confirmation of a positive culture. Only subjects with a confirmed positive culture at baseline were considered evaluable patients for treatment efficacy.

The reported anticipated and unanticipated adverse events (AEs) and serious adverse events (SAEs) associated with the use of the topical foot bath application of the NORS were collected for safety review. Additional safety endpoints were determined by the effect of NORS treatment on oxygen saturation, heart rate and elevation of systemic methemoglobin levels (≤2.5% above baseline), measured pre-, during and post-treatment via a non-invasive co-oximeter. The presence of environmental NOx levels (NO ≤ 25 ppm, NO_2_ ≤ 5 ppm) was also surveyed. Safety endpoints were evaluated in all enrolled participants.

## 3. Results

### 3.1. In Vitro Testing with No-Releasing Gel

#### 3.1.1. Antifungal Time-Kill Assays

**Figure 1:**
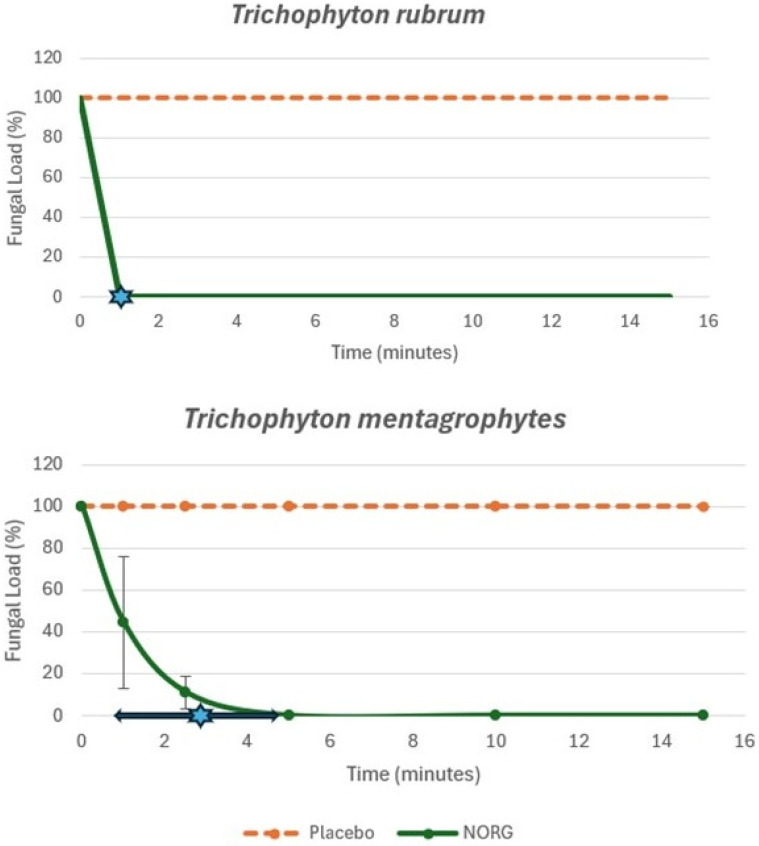
The effect of exposure time on the fungal burden demonstrated a faster mean reduction in fungal burden with the NORG versus the vehicle gel placebo for both fungal species. The mean eradication time (±SD) is represented by the star. Two-tailed, paired *t*-test for *T. rubrum* vs. vehicle placebo: *p* = 0.0041. Two-tailed, paired *t*-test for *T. mentagrophytes* vs. vehicle placebo: *p* = 0.0063.

The NORG demonstrated rapid fungicidal effects. The NORG demonstrated complete fungicidal activity against *T. rubrum* within one minute of exposure in all experimental replicates. Against *T. mentagrophytes*, complete eradication was achieved at an average exposure time of 2.83 min (SD ± 2.02 min). Both fungal species show relatively rapid courses of reduction in fungal loads; the placebo showed no reduction in fungal load over the 15 min time course (Figure 1). It is possible that there may be a fungistatic action of the NORG rather than a fungicidal action; however, no further growth was noted for any of the plated samples post-treatment over the 7-day incubation time course, which would indicate fungistatic action only.

#### 3.1.2. Zone of Inhibition Assays

**Figure 2:**
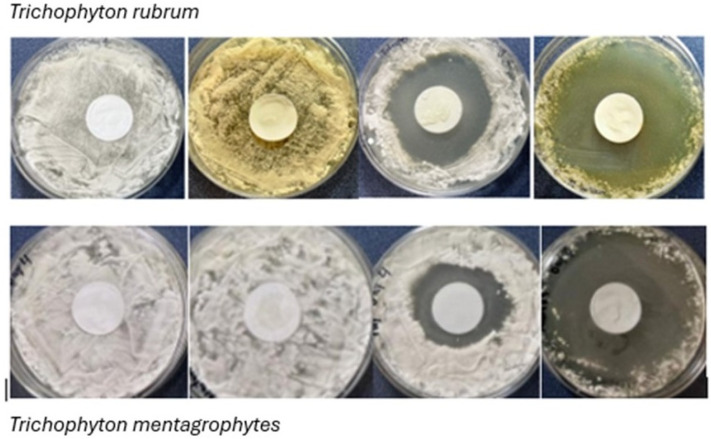
The zone of inhibition assay plates showing representative examples of activity against *Trichophyton rubrum* (**top panel**) and *Trichophyton mentagrophytes* (**bottom panel**). The treatment conditions from left to right are the placebo (vehicle gel), Tolnaftate, Tolcylen, and the NORG.

**Figure 3:**
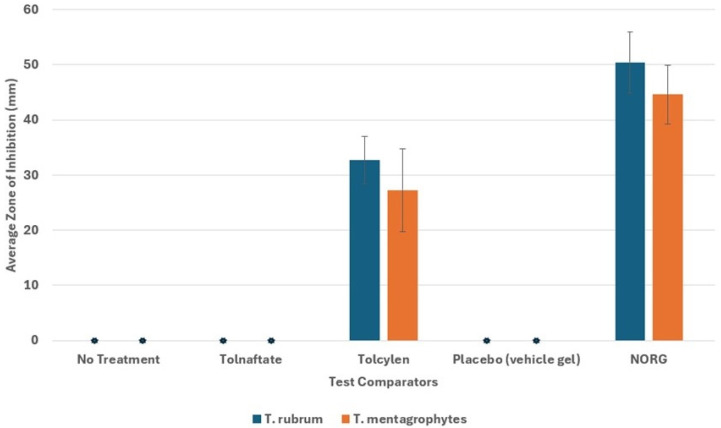
The zone of inhibition assays using synthetic dermal membranes demonstrated an inhibition for NORG and Tolcylen only, with NORG showing significantly larger zones of inhibition than Tolcylen. Two-tailed, paired *t*-test for *T. rubrum*–NORG vs. Tolcylen: *p* = 0.033. Two-tailed, paired *t*-test for *T. mentagrophytes*–NORG vs. Tolcylen: *p* = 0.012.

The NORG penetrates the dermal membrane and retains fungicidal efficacy. Both *T. rubrum* and *T. mentagrophytes* were highly susceptible to the NORG (Figure 2), thereby demonstrating that the NORG effectively penetrated the synthetic dermal membrane without a loss of antifungal action. The NORG averaged a 57% greater antifungal effect compared to Tolcylen (Figure 3), with mean zones of inhibition measured to be 47.6 mm (SD ± 5.6 mm) and 30.3 mm (SD ± 10.5 mm), respectively, at day 8. Tolnaftate failed to produce any zone of inhibition.

#### 3.1.3. Scanning Electron Microscopy

**Figure 4:**
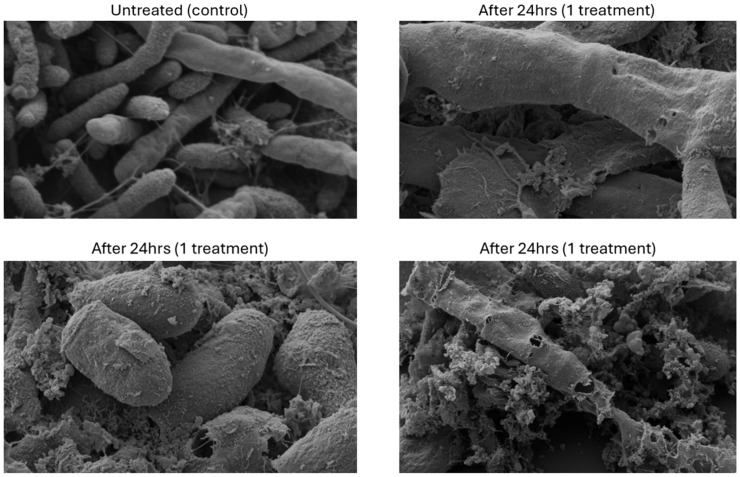
The representative images from the SEM study of *T. rubrum* treated with the NORG (magnification: top left, 5.00 K×; bottom left, 10.00 K×; top right, 10.00 K×; and bottom right, 5.00 K×).

The SEM images revealed dramatic differences in the structural and morphological aspects of *T. rubrum* cells between untreated and NORG-treated samples (Figure 4). The untreated cells showed abundant hyphae and intact fungal envelopes. Treatment with NORG impaired the hyphae formation, disrupted surface morphology, and resulted in visible perforations in the fungal cell walls. Additionally, extensive fungal cell lysis was observed, conclusively demonstrating that NORG acts as a fungicidal rather than merely fungistatic agent. This visual evidence provides a striking demonstration of the mechanism of action for NORG.

A modification of the Franz diffusion cell method through the utilization of an industry-standard synthetic dermal barrier between the antifungal agent and the fungal culture simulated more realistic clinical conditions by evaluating dermal permeation and subsequent antifungal efficacy. This methodological rigor enhances the translational applicability of the findings, suggesting that the NORG could be effectively used in clinical dermatological settings.

### 3.2. In Vivo Pilot Use of No-Releasing Solution for Tinea Pedis

Of the 20 participants enrolled in the study, 13 were randomized to receive the NORS and seven to receive a placebo. Most participants were male (17/20), and the mean age was similar between the NORS and placebo groups (53.2 years and 53.1 years, respectively). There were no significant differences between the active treatment and placebo groups in pre-treatment CSSS (26.3 and 29.7, respectively) or pre-treatment methemoglobin levels (0.3% and 0.2%, respectively). Of the 20 enrolled participants, 12 showed a positive culture and were considered evaluable for efficacy, eight of whom were in the NORS group and four of whom were in the placebo group.

**Figure 5:**
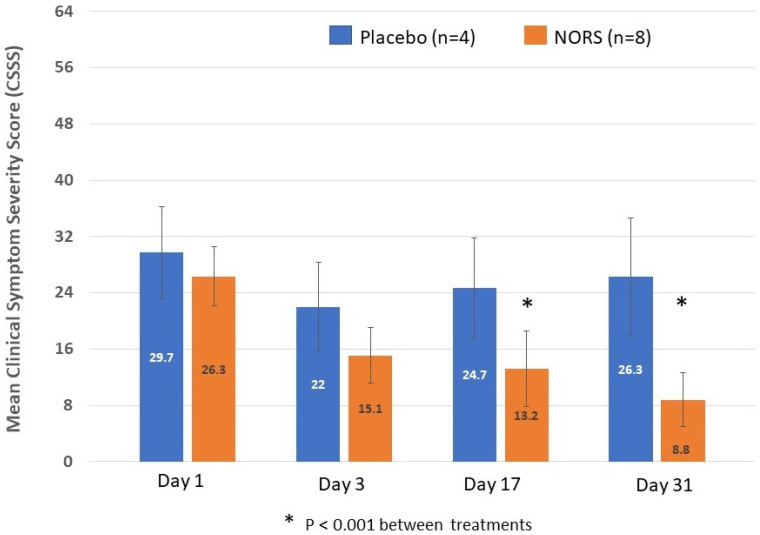
The clinical symptom severity score (CSSS) shows a significant reduction in score for the NORS treatments versus the placebo on day 17 and day 31. Two-way ANOVA was used to test the significance (* = *p* < 0.001).

**Figure 6:**
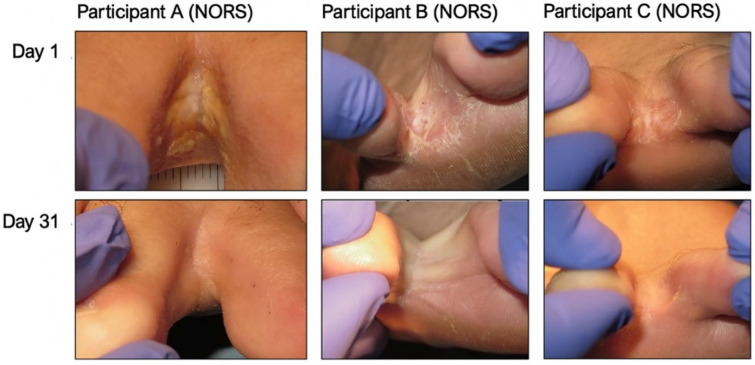
An illustration of clinical improvements seen on day 31 (CSSS < 9) in three NORS patients.

The NORS treatment reduced the CSSS between day 1 and day 31 (4 weeks after the end of treatment) by 17.6 ± 5.7 points on average, which is a decrease of 67% (Figure 5). However, in the placebo group, the CSSS only decreased on average by 3.4 ± 9.0 points, which is 11.4% (*p* < 0.001 for a comparison of decreases for the NORS versus the placebo). There was a continued reduction in the CSSS in the treatment group after the end of the treatment from day 3 to days 17 and 31 (Figure 5). Five NORS participants (5/8 = 62.5%) reached a CSSS < 9 at day 31 (Figure 6) versus 0% (0/4) in the placebo group.

A negative mycological culture was reported on day 31 for 88% (7/8) of participants in the NORS group and 25% (1/4) of participants in the placebo group. All the participants in the NORS group had positive mycological cultures on day 1 and negative mycological cultures on days 3 and 17 for the organisms identified on day 1. At day 31, seven out of eight of the participants remained negative by culture; 1 NORS participant was negative for the original organism but culture-positive with a new organism. All the patients who reached CSSS < 9 also showed a negative KOH/culture. In the placebo group, three out of four of the participants remained culture-positive on days 3, 17, and 31. The remaining placebo participant showed a positive culture at day 17 but was negative by culture on days 3 and 31.

All of the 20 enrolled patients were included in the safety evaluation. The 13 patients who enrolled in the NORS treatment group received a total of 39 NORS footbath treatments. There were no instances of either NO > 25 ppm or NO_2_ > 5 ppm. No participant in either group had an elevated methemoglobin saturation at baseline, and there were no methemoglobin elevations when measured mid-treatment or at treatment completion. A total of 15 AEs were reported by nine participants (14 NORS, 1 placebo; Table 1). No SAEs were reported. There were four AEs in the NORS group that were considered possibly related to the treatment. All four events were mild and transient, resolving without treatment.

## 4. Discussion

NO-releasing formulas have demonstrated potential antifungal effects against dermatophytes in vitro. NO release and in vitro activity of the NORS was confirmed previously against mycelial and spore forms of dermatophytes, while the NORG penetration through the nail plate into the bed was noted in an ex vivo toenail model [18,19] (Table 2). Pilot clinical testing shows evidence of NORS utility for plantar warts and the NORG for onychomycosis [19,20,21]; penetration of NO through the full thickness of the nail plate in vitro was confirmed, in the form of s-nitrosothiol reaction products (Table 2). We now also note the rapid in vitro fungicidal effects of the NORG and the pilot clinical evidence of NORS activity for tinea pedis.

The rising incidence of antifungal resistance necessitates novel therapeutic approaches for dermatophyte infections. The current in vitro data for NO-releasing formulations demonstrate that NO possesses good antifungal activity against the tested dermatophyte strains. The new data presented here for the NORG formulation indicate possible rapid and potent fungicidal activity against both *T. rubrum* and *T. mentagrophytes*. The superior in vitro activity of the NORG versus commercially available Tolcylen and tolnaftate suggests a possible role for the NORG in the treatment of tinea pedis, as with the NORS formulation. The potential for broad-spectrum antifungal efficacy has been noted with NO-releasing formula NVN1000 [23]. Larger-scale clinical trials are required to verify the outcomes of the NORS and the NORG in a wider range of patients and organisms.

SEM provided compelling visual evidence of a possible fungicidal mechanism of the NORG, clearly indicating a disruption of fungal cell structure, impairment of hyphal integrity, and substantial cell-wall perforation. These morphological changes are indicative of severe nitrosative stress induced by nitric oxide, aligning with previously documented mechanisms in the literature [23,24]. This is in contrast to the enzyme-specific target interactions provided by the azole and allylamine antifungals, which disrupt the formation of lanosterol. Each of these antifungal classes targets only a single enzyme, leaving the antifungal activity vulnerable to reduction or disruption by a variety of internal fungal mechanisms or genetic mutations. The multifaceted mechanism of nitric oxide targeting thiol groups, lipid membranes, and nucleic acids may provide a reduced potential for developing antifungal resistance, thereby offering a distinct advantage over traditional single-target antifungal therapies [24,25]. Additionally, nitric oxide’s known role in promoting wound healing through keratinocyte proliferation may confer additional therapeutic benefits, potentially accelerating the restoration of the skin barrier that has been compromised by fungal infection [26]. Future studies providing longer observation are needed to confirm the duration of treatment efficacy, safety, and potential for the development of resistance. The antifungal activity of NO against dermatophytes results from a nitrosative and oxidative reaction, leading to quick NO dissipation; the retention of antifungal activity over time may need to consider retention of reaction intermediate species such as peroxynitrite or s-nitrosothiols rather than NO itself [7,27]; antifungal retention times and duration need confirmation in future research. No evidence for resistance or spontaneous mutation was found in the selected bacteria tested against exogenous NO, but no such testing has been completed for dermatophytes and remains a focus for future research [25].

In the small trial of NORS administered via foot baths for tinea pedis, the treatments were well tolerated and showed good therapeutic efficacy at day 31. A 28-day repeat dose dermal toxicity study in a murine model (OECD-410) and subsequent histological tissue evaluation were completed as part of the commercial NORG product safety substantiation and no toxicity concerns were noted (in-house data). The existing medical literature for topical NO gels, including an alternate commercially available NO-releasing gel for molluscum contagiousum, showed effective transdermal penetration in rats [28] and has not demonstrated a significant risk of toxicity over short-term usage in humans [16,19,20,21,27,29,30,31,32]. No serious adverse events were noted in the large-scale trial of the molluscum contagiousum formula; however, the application-site AEs, such as pain, erythema, pruritis, exfoliation, dermatitis, scar and swelling, were reported in 3.6–18.7% of the test population [16]. The systemic absorption of the NORS for tinea pedis showed no evidence of methemoglobinemia, and phase II research with an NO-releasing gel (SB208 gel containing NVN1000) for tinea pedis found minimal NO absorption with no cases of methemoglobinemia [29]. A short-term in vivo mammalian toxicity study of the SB208 gel (28-day twice daily topical application in miniature swine) showed no macroscopic test article-related findings, other than a transient erythema at the dose application site; additionally, there were no gel-related effects regarding food consumption, body weight, hematology, clinical chemistry and coagulation parameters, ophthalmologic and electrocardiographic measurements, and organ weight [23]. There is no safety data for any NO-releasing formula regarding its use in human pregnancy/lactation/risk of birth defects, fertility, and long-term carcinogenesis. Wider clinical testing and longer-term post-treatment observation are needed within the field of NO-releasing products to provide clinicians with a more complete safety profile.

The NORS tinea pedis trial noted a potential for recurrence up to day 31; a longer-term follow-up is needed to evaluate this potential more thoroughly with respect to antifungal resistance development. Furthermore, it is difficult in dermatophytosis to differentiate recurrence due to reinfection versus resistance. Anecdotal reports from some of the participants noted a reluctance to follow the post-bath foot hygiene recommendations, which resulted in going barefoot and not using new socks/insoles to prevent reinfection. The availability of a topical NORG could represent a more user-friendly formula for therapeutic use versus solution baths; NORG prophylaxis for a period following the NORS baths may also merit further clinical testing.

Dermatophyte skin infections have historically been readily treatable by topical antifungal products, many of which are available for OTC use at a reasonable price. Terbinafine cream is usually the preferred and most potent topical antifungal, but OTC availability is not universal for this product. Moreover, there is increasing evidence of terbinafine resistance in superficial dermatophyte infections, which require alternative treatments. The exploratory results documented here suggest that NO-releasing products may be suitable as one of these alternatives. A more detailed investigation remains ongoing and will be needed to fully document the possible utility of NO-releasing formulas for skin infections.

Regarding onychomycosis, oral therapies are associated with potential for liver dysfunction and drug–drug interactions, which may prevent their use. Topical antifungals are preferred, but no OTC formula is approved for onychomycosis. A resistance to terbinafine is also being noted in onychomycosis treatment. Topical prescription antifungals require long-term use (typically 48 weeks or more), can be expensive to obtain, and clearance rates remain relatively low. The apparent penetration of the NORG into nails places the product well for treating onychomycosis; controlled clinical trials are necessary to provide more systemic evidence of efficacy and safety for this indication. If future explorations confirm antifungal effects in nail infection, the NORG’s mechanisms would be unique in the antifungal spectrum, and OTC availability would also be a widespread benefit for patients.

## 5. Conclusions

Though the current data are limited, these data support the potential of NO-releasing formulations as novel topical treatments for superficial fungal infections. The NO mechanisms of action are hypothesized to provide a lower potential for development of antifungal resistance compared to conventional antifungal therapies. Furthermore, the availability of a topical NORG as an alternative or complement to the NORS foot baths could be beneficial in reducing reinfection in conditions such as tinea pedis. Progression to clinical studies is warranted to confirm the potential clinical uses highlighted in this paper, as well as to further elucidate the safety profile and therapeutic scope of NO-releasing formulations for OTC use.

## Figures and Tables

**Table 1:** The adverse events reported during NORS/placebo treatment of tinea pedis.

Subject	Treatment Group	Adverse Experience	Duration	Severity	Relation to Study Drug	Resolved Yes/No
008	NORS	Sore throat, cough, no fever	2 days	Mild	None	Yes
005	NORS	Sharp pressure on the foot	1–2 min	Mild	Possibly	Yes
005	NORS	The opposite foot is itchy and burning	1 day	Mild	None	Yes
005	NORS	Pre-existing numbness on the ball of the feet and toes	½ day	Mild	None	Yes
004	NORS	Burning sensation on the balls of both feet during tx	15 min	Mild	None	Yes
004	NORS	Low saturation pre-tx; sleep apnea; notified physician	10–20 min	Mild	None	Yes
004	NORS	Itchy feet after using alcohol cleaner during the day	1 day	Mild	None	Yes
003	NORS	A blister on the opposite foot from hiking	1 week	Mild	None	Yes
007	NORS	Environmental NO_2_ alarm; reading 0.5 ppm; transient	5–10 s	Mild	Yes	Yes
024	NORS	Heartburn	1 day	Mild	None	Yes
024	NORS	Soft tissue damage; hit by a car while biking	7 days	Moderate	None	Yes
015	NORS	Pre-study lump on foot; advised to see a physician	Continuous	Mild	None	N/A
015	NORS	Burning ankles; Prior to study	1 day	Mild	Possibly related	Yes
026	NORS	Warm sensation	20–30 min	Mild	Possibly related	Yes
011	Placebo	Upper respiratory tract infection	7 days	Mild	None	No

**Table 2:** Investigation of the commercial NO-Releasing formulas.

	NO-Releasing Solution (NORS)	NO-Releasing Gel (NORG)
In vitro/Ex vivo Testing	• Potent activity against both mycelial and spore forms of *T. rubrum* (ATCC 18758) and *T. mentagrophytes* (ATCC 114841), attributed to NO-mediated antimicrobial mechanisms [18]	• Ex vivo toenail models: the NORG can effectively permeate the nail plate and produce antifungal activity at the site of infection; it achieves penetration of the nail plate and may effectively sterilize the nail bed [19].
	• Robust antifungal activity of NORS against both susceptible and resistant dermatophyte strains, with comparative effectiveness to efinaconazole and terbinafine, even in keratin-rich conditions [22]	• Rapid fungicidal activity against *T. rubrum* (ATCC 28188) and *T. mentagrophytes* (ATCC 18748) within minutes of exposure, effective dermal membrane penetration while retaining antifungal action, and significant morphological damage to fungal cells, indicating a fungicidal activity [See Section 3.1]
Clinical Use	• Plantar warts: ≥70% reduction in the wart area in 21% of patients (NORS 41.1 ppm-min) and 16% (NORS 41.1 ppm-min) after 15 min footbaths 3× weekly for 3 weeks [20]	• Onychomycosis: reports of short-term nightly use show preliminary signs of improvement over short-term follow-up; nail discoloration indicative of NORG nail penetration is seen [19,21]
	• Tinea pedis: significantly reduced CSSS at day 31 for NORS treatment (30 min footbath for 3 consecutive days) versus placebo after 3 consecutive days [see Section 3.2]	

## Data Availability

The original contributions presented in this study are included in the article. Further inquiries can be directed to the corresponding author.
